# Supplementary figures and images for: Bone marrow stromal cells induce an ALDH+ stem cell-like phenotype and enhance therapy resistance in AML through a TGF-β-p38-ALDH2 pathway
Source: PLoS One. 2020 Nov 30;15(11):e0242809. doi: 10.1371/journal.pone.0242809 (PMC7703975; doi:10.1371/journal.pone.0242809)

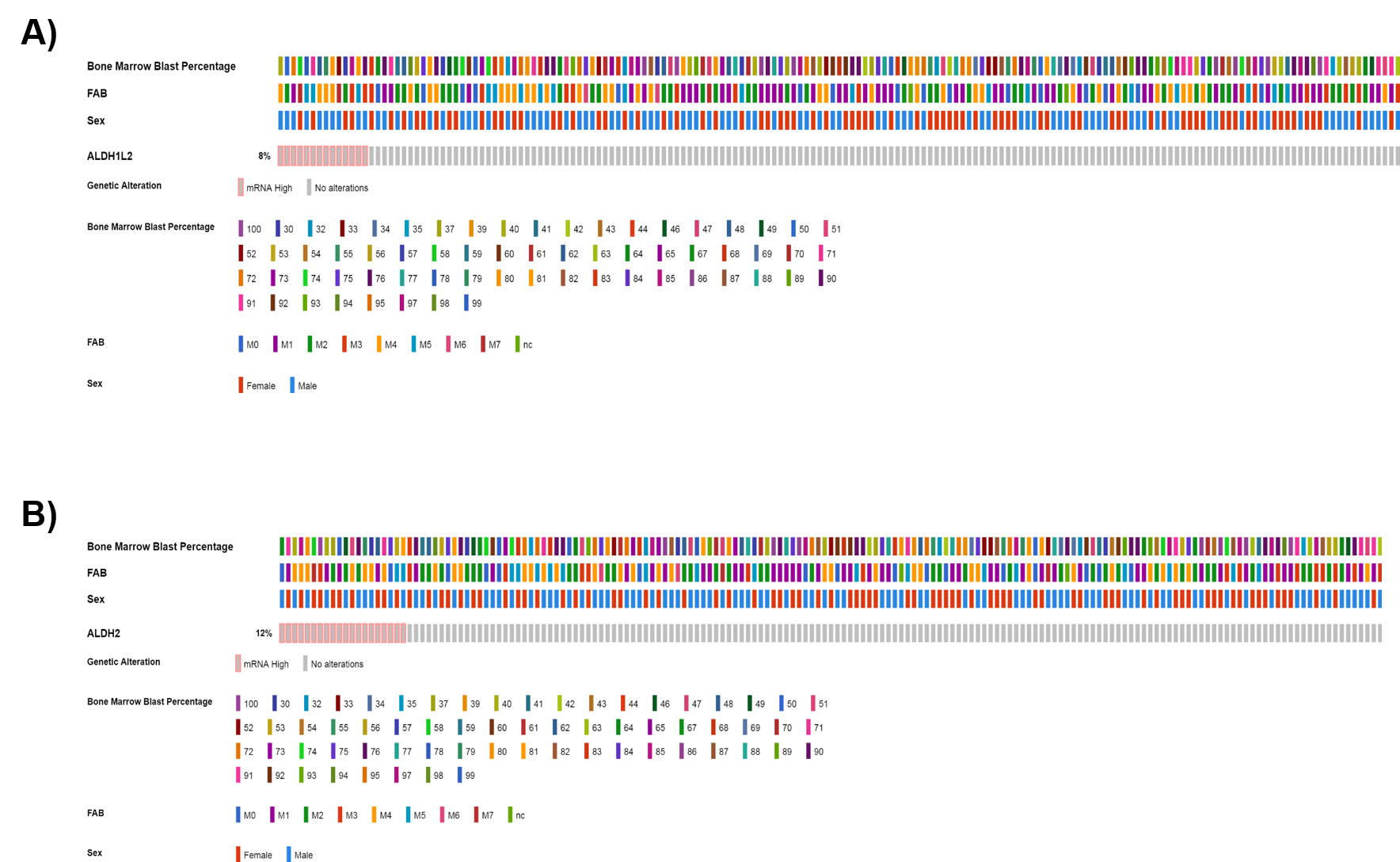

Supplement: S1 Fig — (A) Data extracted from TCGA AML dataset showing mRNA expression levels of ALD1L2 isoform in 8% of AML cases. (B) Data extracted from TCGA AML dataset showing expression of ALDH2 isoform in 12% of AML cases. Figure generated through cBioportal for cancer genomics data analysis tool. (TIF) [file pone.0242809.s001.tif]

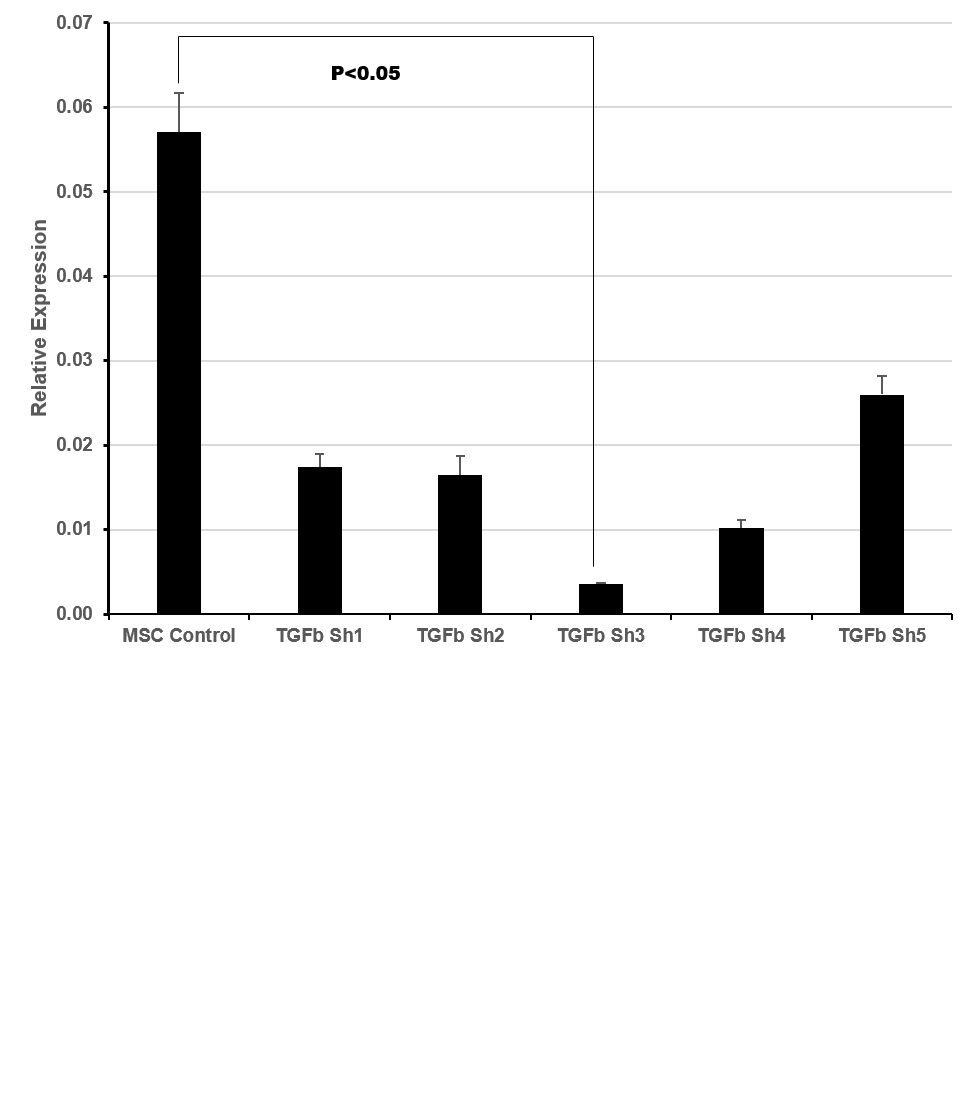

Supplement: S2 Fig — Lentiviral-mediated shRNA was used for stable knockdown of TGF-β1 in human-derived MSCs. TGF-β1 mRNA knockdown efficacy in each shRNA construct was assessed using q-PCR in comparison to control BM-MSCs. (TIF) [file pone.0242809.s002.tif]

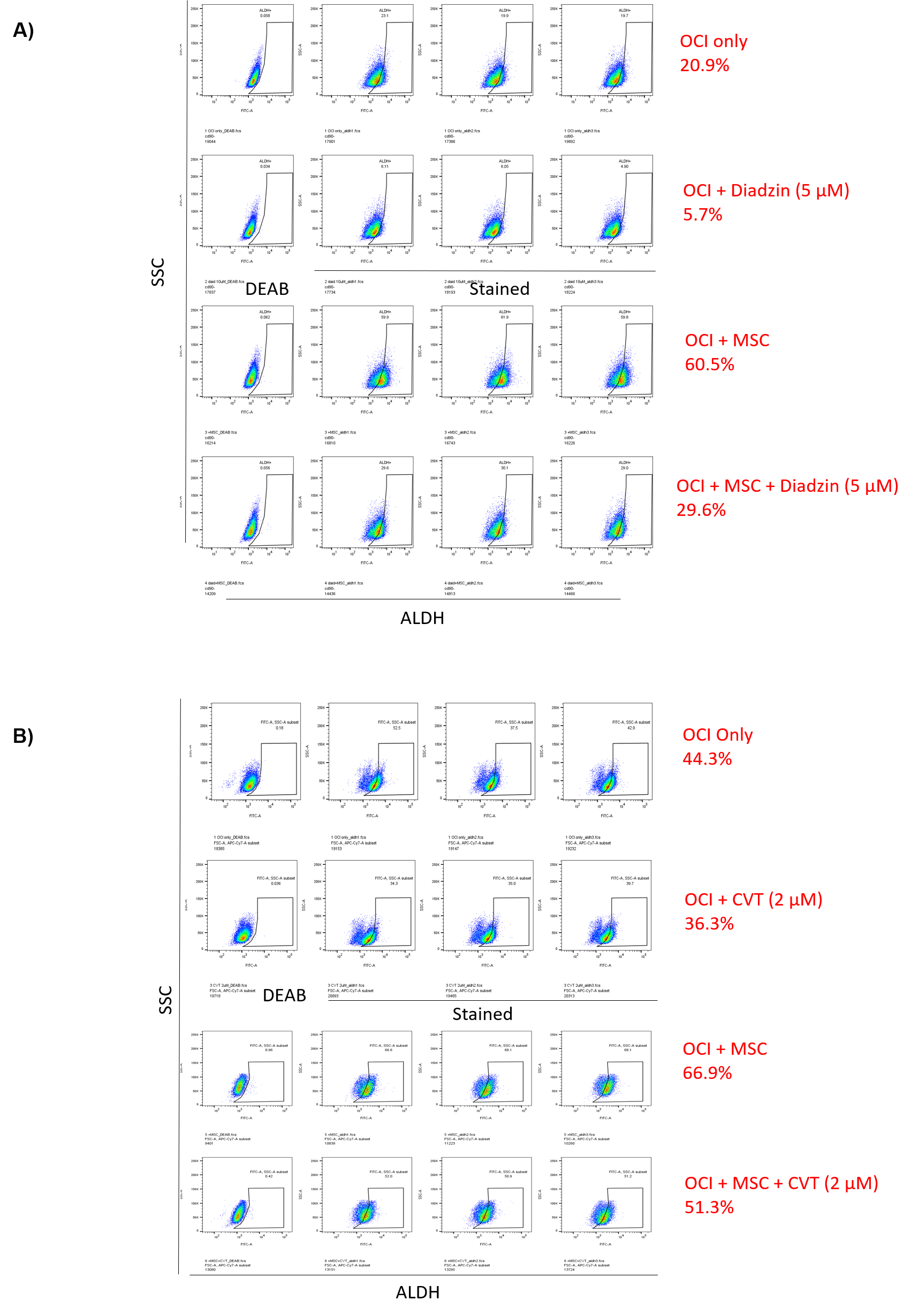

Supplement: S3 Fig — (A) OCI-AML3 cells were cultured with or without BM-MSCs for 3 days. Cells were stained with ALDEFLUOR® and ALDH activity was measured by flow cytometry. Diadzin (5 μM) was added to OCI-AML3 cells alone or co-cultured with BM-MSCs, and ALDH activity was compared between treated and untreated cells. (B) The same experiment was performed as in A, using CVT-10216 instead of diadzin. Dot plots shown here were used to generate bar graphs in Fig 5. (TIF) [file pone.0242809.s003.tif]

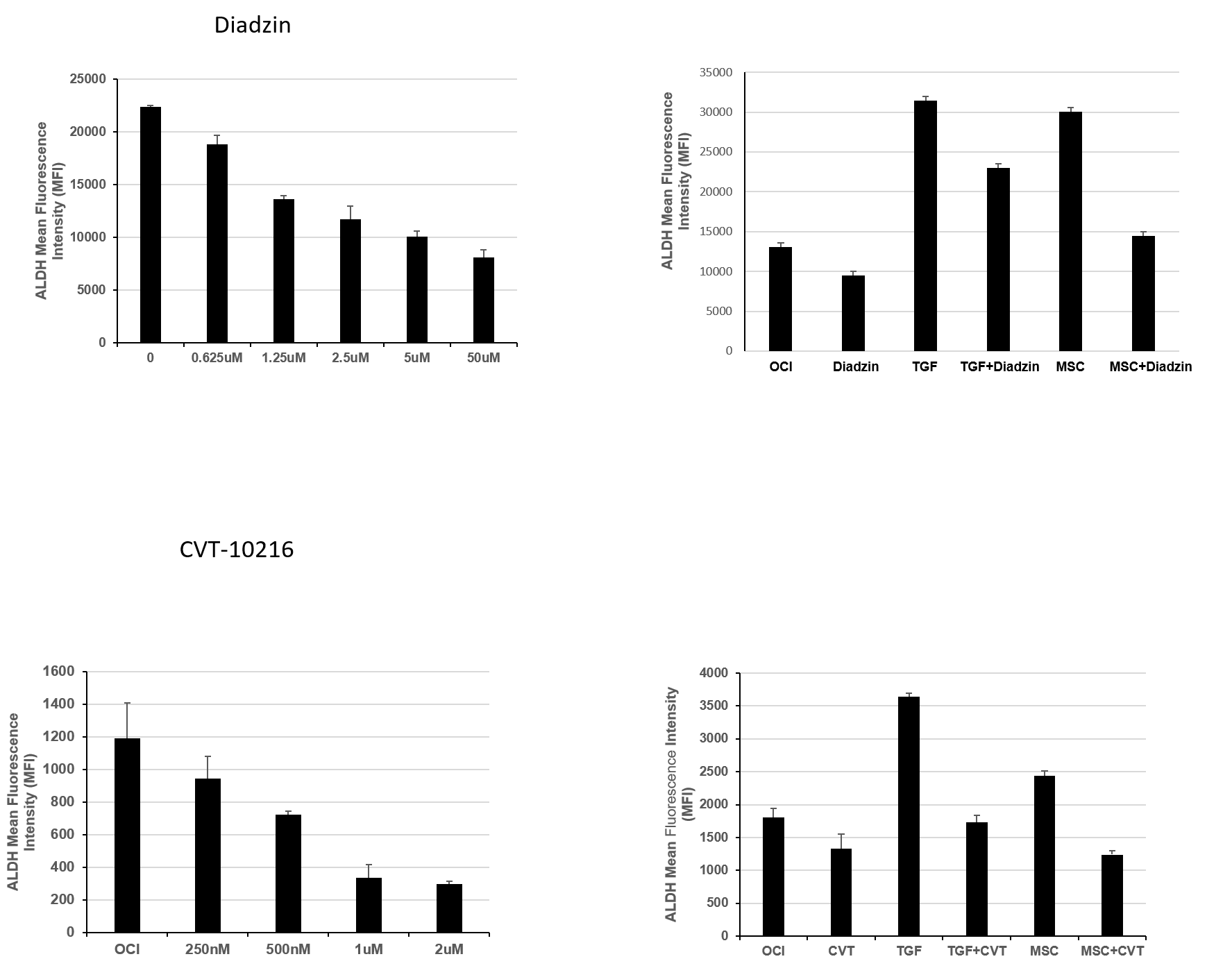

Supplement: S4 Fig — (TIF) [file pone.0242809.s004.tif]

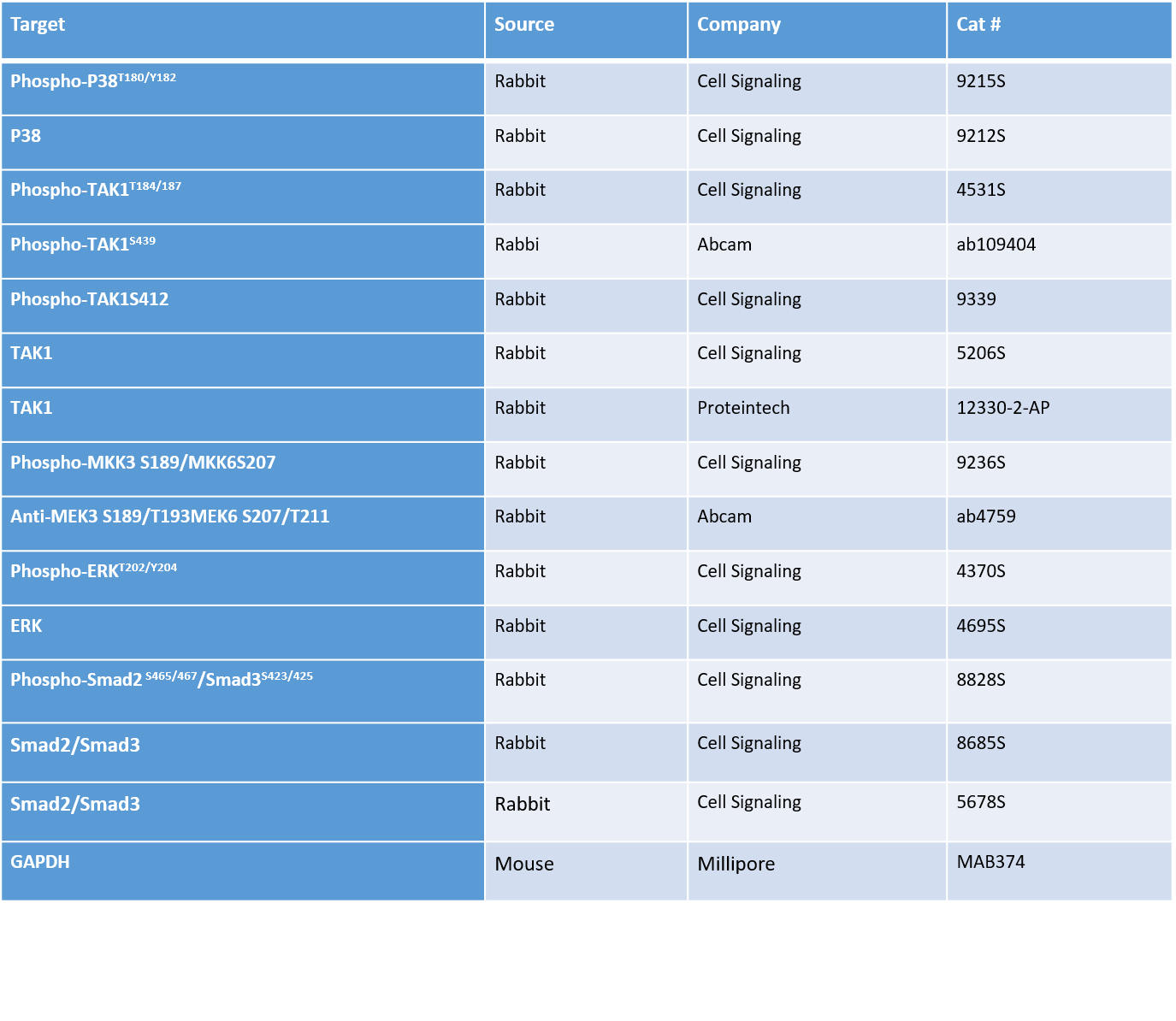

Supplement: S1 Table — List of antibodies used for Western blot analysis of protein expression of downstream targets of the TGF-β signaling pathways. (TIF) [file pone.0242809.s005.tif]

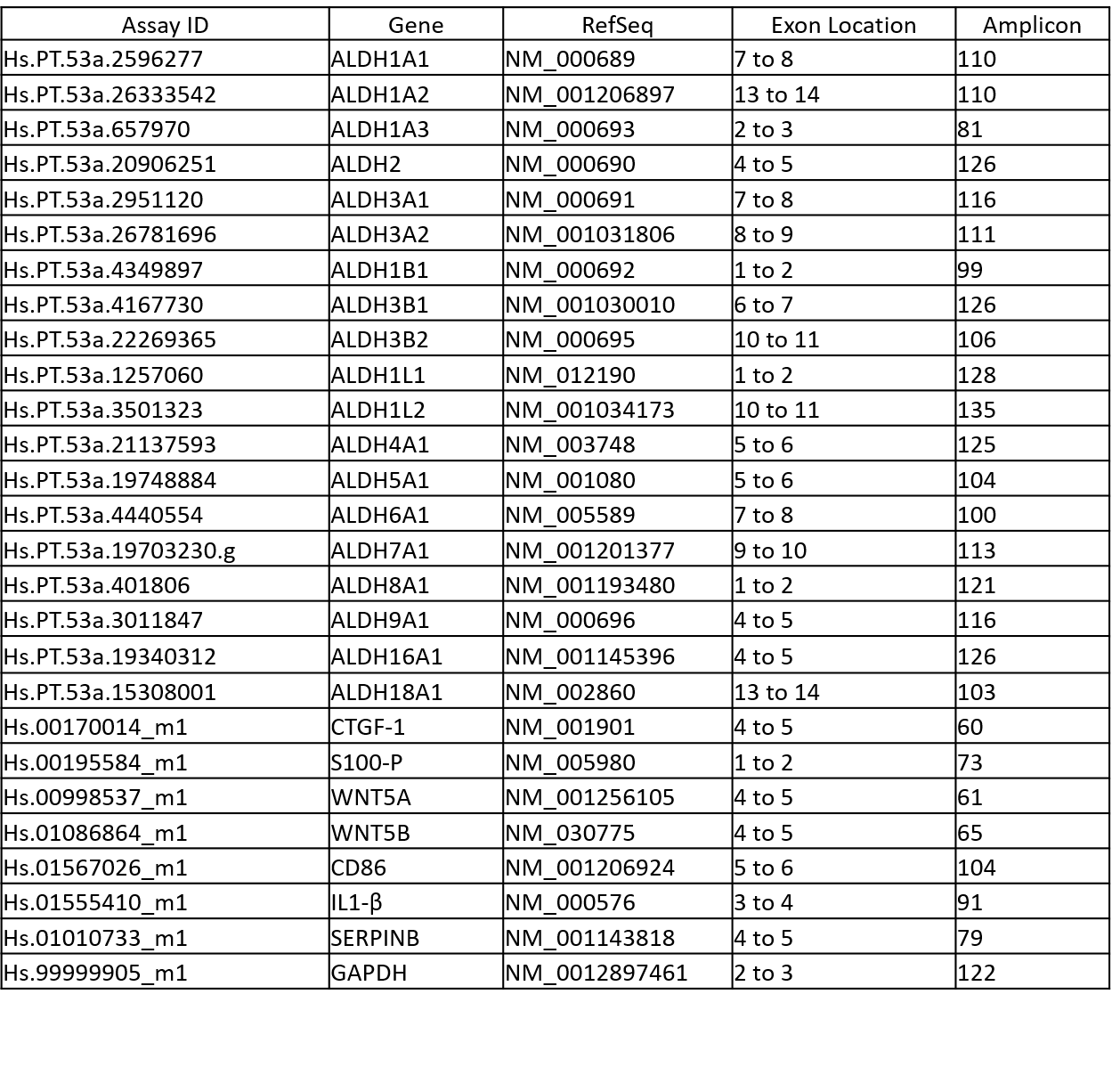

Supplement: S2 Table — List of primers used to analyze RNA expression of different ALDH isoforms as well as other TGF-β downstream targets by RT-PCR. (TIF) [file pone.0242809.s006.tif]

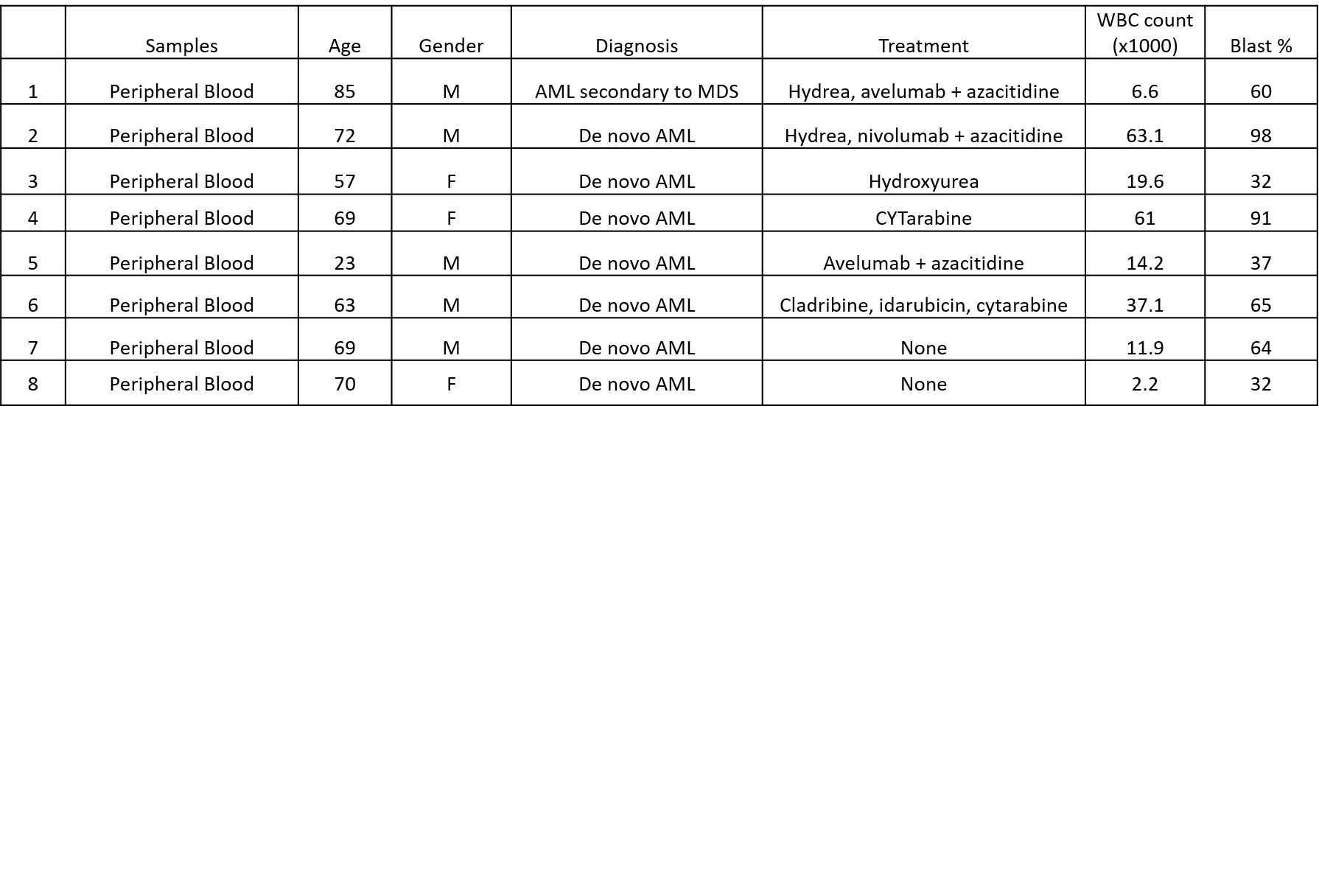

Supplement: S3 Table — Patient demographics and clinical parameters corresponding to the 8 AML peripheral blood patient samples. (TIF) [file pone.0242809.s007.tif]
