# Supplementary material for: Bone marrow stromal cells induce an ALDH+ stem cell-like phenotype and enhance therapy resistance in AML through a TGF-β-p38-ALDH2 pathway
Source: PLoS One. 2020 Nov 30;15(11):e0242809. doi: 10.1371/journal.pone.0242809 (PMC7703975; doi:10.1371/journal.pone.0242809)
Supplement: S1 Raw images — (PDF) [file pone.0242809.s008.pdf]

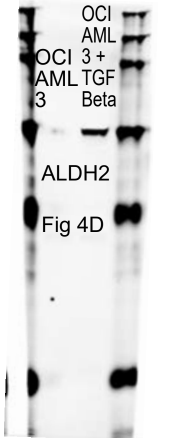
OCI AML 3
OCI AML 3 + TGF Beta
ALDH2
Fig 4D

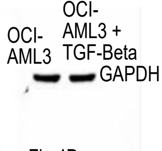

Fig 4D

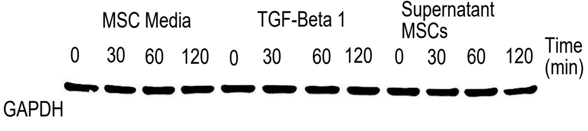
MSC Media
TGF-Beta 1
Supernatant MSCs
0
30
60
120
0
30
60
120
0
30
60
120
Time (min)
GAPDH

Fig 4E

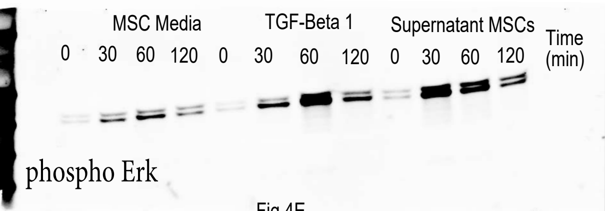
MSC Media
TGF-Beta 1
Supernatant MSCs
Time (min)
0 30 60 120 0 30 60 120 0 30 60 120
phospho Erk

Fig 4E

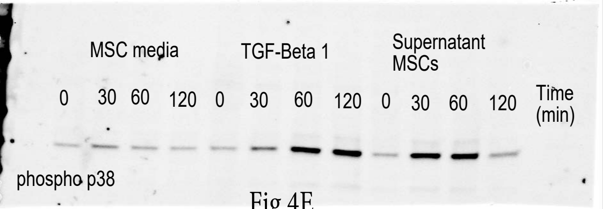
MSC media
TGF-Beta 1
Supernatant MSCs
0 30 60 120 0 30 60 120 0 30 60 120
Time (min)
phospho p38
Fig 4E

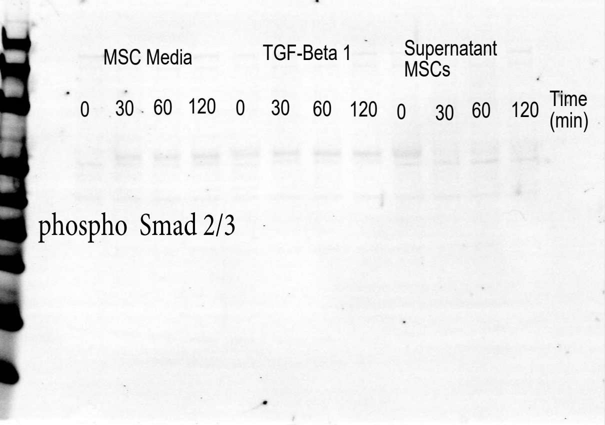
MSC Media
TGF-Beta 1
Supernatant MSCs
0
30
60
120
0
30
60
120
0
30
60
120
Time (min)
phospho Smad 2/3

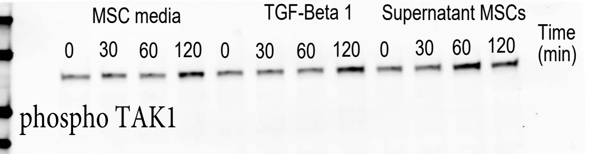
MSC media
TGF-Beta 1
Supernatant MSCs
Time (min)
0 30 60 120 0 30 60 120 0 30 60 120
phospho TAK1

Figure 4E

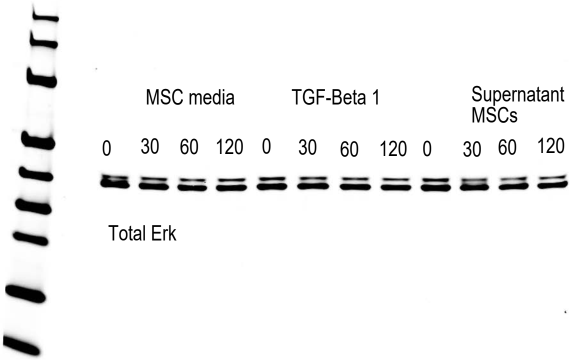
MSC media
TGF-Beta 1
Supernatant MSCs
0
30
60
120
0
30
60
120
0
30
60
120
Total Erk

Figure 4E

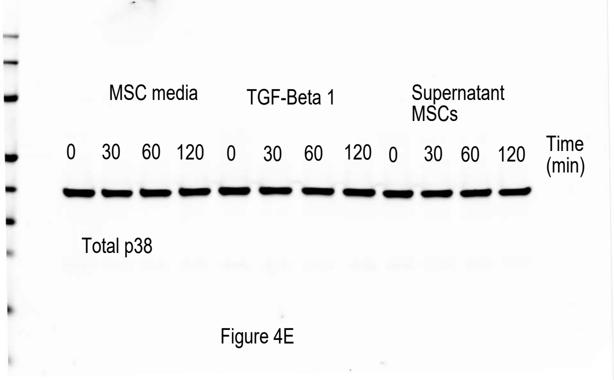
MSC media
TGF-Beta 1
Supernatant MSCs
0 30 60 120 0 30 60 120 0 30 60 120
Time (min)
Total p38

Figure 4E

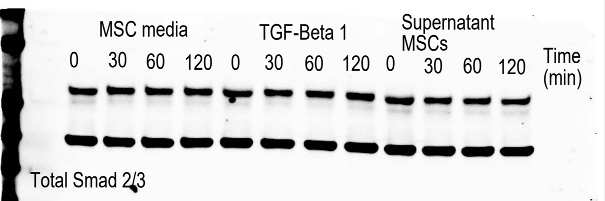
MSC media
TGF-Beta 1
Supernatant MSCs
0
30
60
120
0
30
60
120
0
30
60
120
Time (min)
Total Smad 2/3

Figure 4E

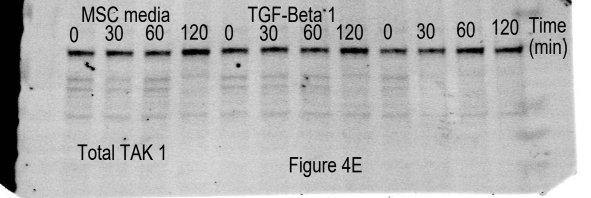
MSC media
TGF-Beta 1
0 30 60 120 0 30 60 120 0 30 60 120 Time (min)
Total TAK 1
Figure 4E

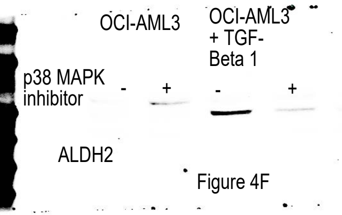

Figure 4F

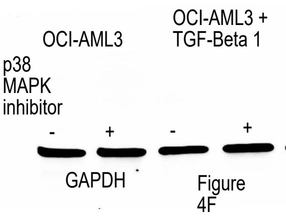

Figure 4F

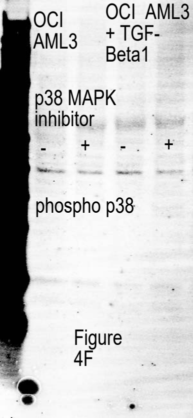
OCI AML3
OCI AML3 + TGF-Beta1
p38 MAPK inhibitor
- + - +
phospho p38
Figure 4F

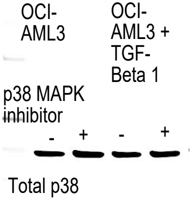

Figure 4F
