# Supplementary material for: Bone marrow stromal cells induce an ALDH+ stem cell-like phenotype and enhance therapy resistance in AML through a TGF-β-p38-ALDH2 pathway
Source: PLoS One. 2020 Nov 30;15(11):e0242809. doi: 10.1371/journal.pone.0242809 (PMC7703975; doi:10.1371/journal.pone.0242809)
Supplement: Attachment — Submitted filename: Response to Reviewers.docx [file pone.0242809.s009.docx]

Section of Molecular Hematology and Therapy, Department of Leukemia

The University of Texas MD Anderson Cancer Center

1515 Holcombe Blvd Houston Texas, 77030

Joerg Heber, Ph.D.

Editor-in-Chief, *PLoS*One

October 23, 2020

Dear Dr. Heber,

I am writing to you in reference to our manuscript entitled “Bone marrow stromal cells induce an ALDH^+^ stem cell-like phenotype and enhance therapy resistance in AML through a TGF-β-p38-ALDH2 pathway” (reference number: PONE-D-20-26977), which was recently peer-reviewed.

First and foremost, I would like to thank you and the reviewers, on behalf of all authors of the manuscript, for the time and effort that you spent reviewing our work. Your comments have been well received and greatly appreciated, as we believe every single point raised contributes towards improving the quality of our manuscript. I have thoroughly discussed all points with my co-authors and we consequently made appropriate adjustments to our manuscript to address the reviewers’ concerns. We believe our revised manuscript now meets *PLoS*One publication criteria and requirements.

Please proceed to the following pages of this letter, where we provide a detailed point-by-point response to the comments and concerns that were raised, highlighting adjustments that were made to address them.

Once again, thank you for considering our work for publication at your esteemed journal.

Sincerely,

Venkata Lokesh Battula, Ph.D.

**Response to comments**

**Academic editor comments**:

**1.** **Please ensure that your manuscript meets PLOS ONE's style requirements, including those for file naming.**

We have carefully reviewed PLOS ONE manuscript formatting guidelines and used the provided templates to ensure compliance with all submission guidelines. Font changes, formatting, and title page adjustments were made accordingly. Additionally, we have changed the figures layout, separated each figure into a separate file, and converted all files into tiff format. We renamed all submitted files according to journal requirements. PACE tool was used to ensure adherence of our figures to the recommended guidelines

**2. Please move Figure S1, describing the results of animal experiments, into the main figures.**

We reorganized our figures to an arrangement that includes 6 main figures instead of the previous 4 figures. Figures that include important findings, such as figure S1 describing results of animal experiments, have been moved from supplementary data to main figures. Please note that what was previously figure S1 has now been moved to figure 1A-B. Accordingly, we made the needed changes to figure captions as well as in-text citations to match the new figure arrangement.

**3. Please provide additional details regarding participant consent. In the ethics statement in the Methods and online submission information, please ensure that you have specified (1) whether consent was informed and (2) what type you obtained (for instance, written or verbal, and if verbal, how it was documented and witnessed). If your study included minors, state whether you obtained consent from parents or guardians.**

In our study, written informed consent was obtained from all healthy individual human MSC donors according to the Declaration of Helsinki. Our study did not include any minors. This information is now added under ‘ethics approval and consent to participate’ in the methods section of the revised manuscript.

**4. Please provide additional information about the HEK293T cell line used in this work, including source, history, culture conditions and any quality control testing procedures (authentication, characterization, and mycoplasma testing).**

HEK293T cells were purchased from ATCC^®^ (Cat# CRL-3216). Cells were cultured in Dulbecco’s Modified Eagle’s Medium (DMEM) high glucose formulation purchased from Media Tech, Inc supplemented with 10% Fetal Bovine Serum (FBS) purchased from Gibco, and 1% Penicillin/Streptomycin. HEK293T are tested routinely in our laboratory for mycoplasma contamination every 4-6 months. All tests for mycoplasma contamination turned out negative. Please note that this information has been added to the methods section under ‘Cell culture of primary MSCs, leukemia cell lines, and patient samples’.

**5. In your Methods section, please provide additional information about the participant recruitment method for the isolation of human MSCs from healthy patient donors. Please ensure you have provided sufficient details to replicate the analyses such as: a) the recruitment date range (month and year), b) a table of relevant demographic details, c) a description of how participants were recruited, and d) descriptions of where participants were recruited and where the research took place.**

Human MSC donors were recruited over a time period between January, 2014 and December, 2019. All donors were healthy individuals between the ages 25-45. All healthy patient donors were recruited according to a protocol approved by MD Anderson Cancer Center institution review board (IRB). Healthy patient donors undergoing bone marrow harvest for allogeneic stem cell transplantation were recruited. All participants provided written informed consent as per the declaration of Helsinki. Participants were recruited and bone marrow samples were collected at the Stem Cell Transplantation and Cellular Therapy Department at MD Anderson Cancer Center.

**6. We note that you have indicated that data from this study are available upon request. PLOS only allows data to be available upon request if there are legal or ethical restrictions on sharing data publicly.**

**In your revised cover letter, please address the following prompts:**

**a) If there are ethical or legal restrictions on sharing a de-identified data set, please explain them in detail (e.g., data contain potentially sensitive information, data are owned by a third-party organization, etc.) and who has imposed them (e.g., an ethics committee). Please also provide contact information for a data access committee, ethics committee, or other institutional body to which data requests may be sent.**

**b) If there are no restrictions, please upload the minimal anonymized data set necessary to replicate your study findings as either Supporting Information files or to a stable, public repository and provide us with the relevant URLs, DOIs, or accession numbers..**

Please note that we have changed our data availability statement to indicate that all data based on which we make our conclusions in this manuscript are available to the public without any restrictions. We have submitted all supplementary data as individual files. There are neither legal nor ethical restrictions on public sharing of data generated in this research work, therefore, prompt **a** is not applicable to our study.

For prompt **b,** we have uploaded our RNA sequencing data to support our findings that TGF-β-associated gene signature is activated in AML cells in the presence of MSCs to a publically available database on NCBI. The accession number for our RNA sequencing dataset is: GSE152996 and is accessible to the public at the following URL: https://urldefense.com/v3/__https://www.ncbi.nlm.nih.gov/geo/query/acc.cgi?acc=GSE152996__;!!PfbeBCCAmug!0C2vyQN-voc9eR8fp-MRw64m1VA7U52_H7qqRNtcfY-BluUKDjg3RMAYRcWIeDES0UM$

All other supplementary data have been submitted as individual supporting information files. The aforementioned adjustments have been included in the revised version of our cover letter.

**7. PLOS ONE now requires that authors provide the original uncropped and unadjusted images underlying all blot or gel results reported in a submission’s figures or Supporting Information files.**

Original uncropped and unadjusted western blot images have been provided as supporting information files. A sentence has been added to our revised cover letter to reflect this change.

**8. Your ethics statement should only appear in the Methods section of your manuscript. If your ethics statement is written in any section besides the Methods, please delete it from any other section.**

In the revised manuscript, our ethics statement appears only at the end of our methods section. It has been deleted from other sections of the manuscript.

**9. Please include captions for your Supporting Information files at the end of your manuscript, and update any in-text citations to match accordingly.**

Please note that we have added captions for supporting information at the end of the manuscript under a new section ‘Supporting information’. In-text citations are also updated to match our new figure arrangement.

**Reviewers’ Comments:**

**Reviewer #1:**

1. **Abstract section must be revised, it must be more organic**

Thank you for your comments. Please note that we have re-written the abstract of our manuscript to include more details about our study and its aim. We have reviewed our abstract several times to improve its quality.

2. **Regarding apoptosis evaluation, the authors must better explain the results obtained. Did the cells die from necrosis, apoptosis (early or late apoptosis)?**

We have evaluated treatment-induced cell death in OCI-AML3 cells by flow cytometry using both Annexin V and DAPI staining. We found that the majority of cells were Annexin V^+^/ DAPI^+^ , indicating that the predominant mechanism of treatment-induced cell death in OCI-AML3 cells is late apoptosis as opposed to early apoptosis and/or necrosis. In the revised manuscript, we included a more elaborate explanation of these findings in the results section under ‘ALDH2 inhibitors sensitize AML cells to standard chemotherapy’, in addition to addressing this question in the our revised discussion section

3. **The figure 4D must be discuss in Discussion section. Its explanation is reported only in figure legend. Simplified schematic representation of the interaction between MSCs and AML cells must be fully explained in this section.**

The revised discussion section in our manuscript now includes a paragraph which extensively discusses the findings portrayed in our simplified schematic diagram. What was previously figure 4D is now figure 6D in the new figure arrangement. Reference to this figure can be found at the end of our results section as well as the summary statement of our revised discussion.

4. **The English language must be revised.**

Our manuscript has been subjected to multiple rounds of editing for sentence structure, grammatical errors, typing mistakes, and overall flow of writing. Both corresponding authors read and edited the manuscript multiple times until we wrote our final version of the manuscript. The final version was then sent to all co-authors who also read the manuscript and suggested edits. Additionally, at MD Anderson Cancer Center, there is a specialized department for scientific editing whereby expert scientific writers provide comments and advice to improve manuscripts prior to journal submission. We sent the final version of our manuscript to that department. We received their feedback and implemented all their suggested changes prior to submitting our work to PLOS ONE.

**Reviewer #2:**

1. **The authors should re-write the abstract section. Abstract should better explain the aim of the study.**

Thank you for your comments. We have reviewed our abstract and we re-wrote it to include sufficient details and explanations pertaining to the aim of our study.

2. **The authors should re-write the discussion section. In the discussion section, the authors should enlarge the discussion of the different experiments that were done.**

Please note that we have extensively revised our discussion section, we expanded the discussion to include sections that address specific experiments described in the results section of our manuscript. We also broadened the scope of the discussion and included additional references.
